# Cortical GABAergic neurons are more severely impaired by alkalosis than acidosis

**DOI:** 10.1186/1471-2377-13-192

**Published:** 2013-12-05

**Authors:** Shuyan Zhang, Piyun Sun, Zhongren Sun, Jingyu Zhang, Jinlong Zhou, Yingli Gu

**Affiliations:** 1 Department of Neurology, The Fourth Affiliated Hospital of Harbin Medical University, 37 Yiyuan Street, Harbin 150001, P.R. China; 2 Heilongjiang University of Chinese Medicine, Harbin, Heilongjiang 150040, China

**Keywords:** Acidosis, Alkalosis, Neuron, Synapse, Action potential, Synaptic potential and cortex

## Abstract

**Background:**

Acid–base imbalance in various metabolic disturbances leads to human brain dysfunction. Compared with acidosis, the patients suffered from alkalosis demonstrate more severe neurological signs that are difficultly corrected. We hypothesize a causative process that the nerve cells in the brain are more vulnerable to alkalosis than acidosis.

**Methods:**

The vulnerability of GABAergic neurons to alkalosis versus acidosis was compared by analyzing their functional changes in response to the extracellular high pH and low pH. The neuronal and synaptic functions were recorded by whole-cell recordings in the cortical slices.

**Results:**

The elevation or attenuation of extracellular pH impaired these GABAergic neurons in terms of their capability to produce spikes, their responsiveness to excitatory synaptic inputs and their outputs via inhibitory synapses. Importantly, the dysfunction of these active properties appeared severer in alkalosis than acidosis.

**Conclusions:**

The severer impairment of cortical GABAergic neurons in alkalosis patients leads to more critical neural excitotoxicity, so that alkalosis-induced brain dysfunction is difficultly corrected, compared to acidosis. The vulnerability of cortical GABAergic neurons to high pH is likely a basis of severe clinical outcomes in alkalosis versus acidosis.

## Background

Brain functions are fulfilled based on encoding analogue signals at the synapses and digital spikes at the nerve cells [1-3]. The processing of these brain codes has been found to be impaired during the neurological disorders, e.g., epilepsy, ischemia and neurodegeneration [4-8]. However, the pathological features of neuronal and synaptic encodings in acid–base imbalance remain unclear. In addition, the patients suffered from alkalosis demonstrated severer brain dysfunction and alkalosis-induced neuropsychological deficits were difficultly corrected, compared with acidosis [9-12]. We hypothesized that the functions of the neurons and the synapses in the central nervous system might be more vulnerable to alkalosis than acidosis.

To test this hypothesis, we analyzed the functional changes of GABAergic neurons in response to alkalosis versus acidosis by electrophysiological approach in cortical slices. The neuronal functions in our analysis included their active properties, such as their capability to produce spikes, their responsiveness to excitatory synaptic inputs as well as their output of inhibitory synapses. The analyses of these parameters are based on a fact that these active properties are modulated by the intracellular biochemical reactions, in which the activity of the enzymes is sensitive to pH in the internal environment. The use of GABAergic neurons to analyze neural vulnerability is based on the facts that they are sensitive to pathological factors [6,8,13-15], and that they make excitatory neurons to be coordinately working in neural networks [16,17].

## Methods

### Brain slices and neurons

The entire procedures were approved by the Institutional Animal Care and Use Committee in Heilongjiang, China. The cortical slices (400 μm) were made from FVB-Tg(Gad-GFP)4570 Swn/J mice (Jackson Lab, USA) in postnatal day 18 ~ 22. Mice were anesthetized by inhaling isoflurane and decapitated by a guillotine. The slices were cut by a Vibratome in oxygenated (95% O_2_ and 5% CO_2_) artificial cerebrospinal fluid (ACSF), in which the concentrations (mM) of different components were 124 NaCl, 3 KCl, 1.2 NaH_2_PO_4_, 26 NaHCO_3_, 0.5 CaCl_2_, 4 MgSO_4_, 10 dextrose, and 5 HEPES, pH 7.35 at 4°C. The slices were held in (95% O_2_ and 5% CO_2_) ACSF (124 NaCl, 3 KCl, 1.2 NaH_2_PO_4_, 26 NaHCO_3_, 2.4 CaCl_2_, 1.3 MgSO_4_, 10 dextrose, and 5 HEPES, pH 7.35) at 25°C for two hours. A slice was transferred to a submersion chamber (Warner RC-26G) that was perfused with ACSF oxygenated at 31°C for whole-cell recording [8,18-21]. Chemical reagents were from Sigma.

Cortical GFP-labeled GABAergic neurons in layer II-III of the sensory cortices were selected for whole-cell recording under DIC-fluorescent microscope (Nikon, FN-E600, Japan), in which an excitation wavelength was 488 nm. The neurons showed fast spiking with no the adaptation in spike amplitudes and frequency, typical properties for interneurons [16,17,22,23].

### In vitro models of cellular alkalosis and acidosis

Cellular alkalosis and acidosis were simulated by changing the pH environment for the cortical tissues, in which we perfused ACFS with alkalinization (pH 8.0) or acidification (pH 6.5) onto the brain slices following control ACSF. The components in these solutions were identical except for pH. Neuronal functions were recorded initially in control ACSF for 15 minutes, and then recorded in the ACSF with alkalinization (neuronal alkalosis) or acidification (neuronal acidosis), i.e., a sequence from control to alkalosis or acidosis.

### Whole-cell recording and neuronal functions

The neurons were recorded by an AxoPatch-200B amplifier under the conditions of the voltage-clamp for their synaptic activities and the current-clamp for their active intrinsic property. Electrical signals were inputted into pClamp 10 (Axon Instrument Inc USA) for data acquisition and analyses. The output bandwidth in this amplifier was 3 kHz. The pipette solution for studying excitatory events included (mM) 150 K-gluconate, 5 NaCl, 5 HEPES, 0.4 EGTA, 4 Mg-ATP, 0.5 Tris-GTP, and 5 phosphocreatine (pH 7.35); [24,25]. The solution for studying inhibitory synapses contained (mM) 130 K-gluconate, 20 KCl, 5 NaCl, 5 HEPES, 0.5 EGTA, 4 Mg-ATP, 0.5 Tris–GTP and 5 phosphocreatine [26-28]. The pipette solutions were freshly made and filtered (0.1 μm). The osmolarity was 295 ~ 305 mOsmol, and pipette resistance was 5 ~ 6 MΩ.

The functions of GABAergic neurons assessed in our study included their active properties, such as their capability to produce spikes, their responsiveness to excitatory synaptic inputs and their output by inhibitory synapses [8]. Their responses to excitatory synaptic inputs were measured by the whole-cell voltage-clamp recording, in which spontaneous excitatory postsynaptic currents (sEPSC) were recorded at these GABAergic neurons [8,29] in presence of 10 μM bicuculline. It is noteworthy that sEPSCs were blocked by using 10 μM 6-Cyano-7-nitroquinoxaline-2,3-(1H,4H)-dione onto the cortical slices at the end of experiments, i.e., these synapses are glutamatergic.

The response of pyramidal neurons to inhibitory synapses was assessed by recording spontaneous inhibitory postsynaptic currents (sIPSC). 10 μM CNQX and 40 μM D-amino-5-phosphonovanolenic acid were added into the ACSF to block ionotropic glutamate receptors and to isolate IPSCs out [26]. 10 μM bicuculline was washed onto these slices at the end of experiments to examine whether synaptic responses were mediated by GABA_A_R, which did block sIPSCs in our experiments. Series and input resistances for all of the neurons were monitored by injecting hyperpolarization pulses (5 mV/50 ms), and calculated by voltage pulses vs. instantaneous and steady-state currents.

**Figure 1:**
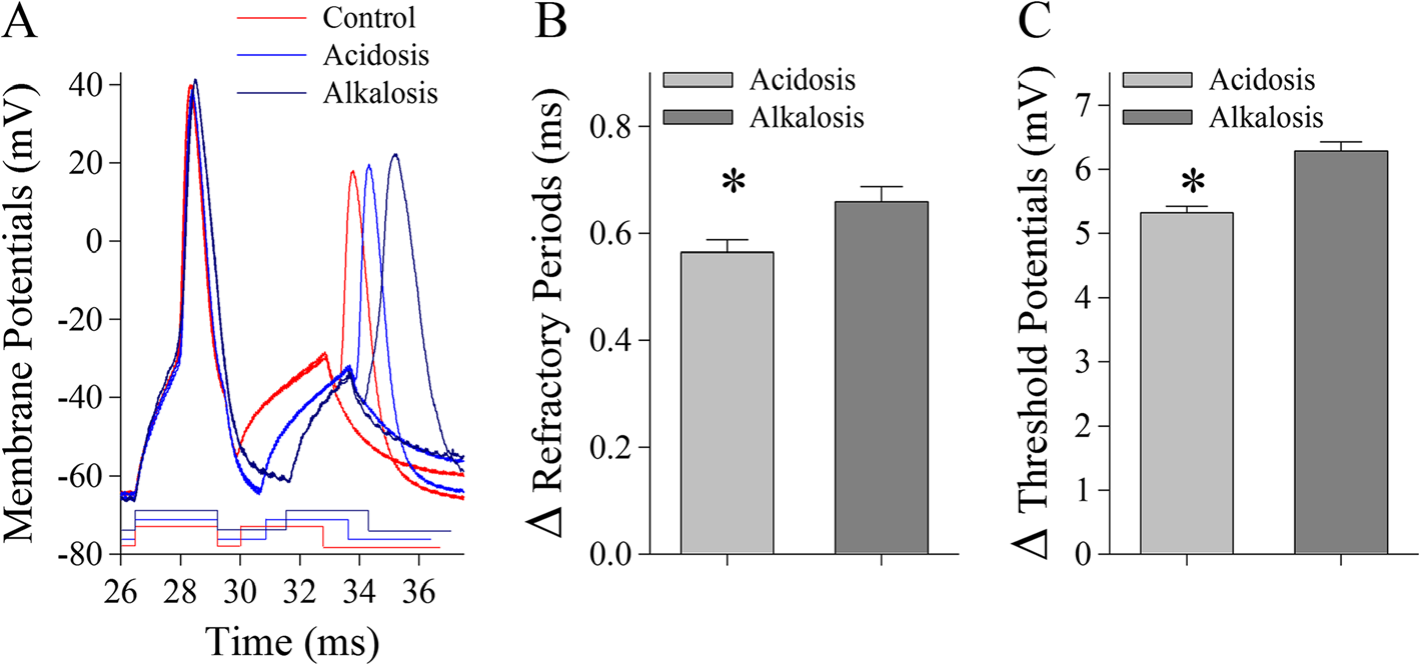
**The spike encoding processes of cortical GABAergic neurons are more vulnerable to alkalosis than acidosis by their influence on refractory periods and threshold potentials mediated by voltage-gated sodium channels. A)** Superimposed waveforms show refractory periods measured by identical protocols under the conditions of control (red trace), acidosis (blue) and alkalosis (dark-blue). **B)** shows statistical results in the net changes of spike RPs under the conditions of acidosis (gray bar) and alkalosis (dark-gray, an asterisk, p < 0.05; n = 15). **C)** illustrates ΔVts under the conditions of acidosis (gray bar) and alkalosis (dark-gray, an asterisk, p < 0.05; n = 15).

Action potentials at GABAergic neurons were induced by injecting depolarization pulses, whose intensity and duration were changed based on the aim of experiments. The ability of producing sequential spikes was evaluated by measuring inter-spike intervals (ISI) when the depolarization pulse (200 ms) was given before and after acidosis or alkalosis, as well as by measuring stimulus intensities versus number of spikes (input-out-curve). These parameters denoted the ability of the neurons to convert excitatory inputs into spikes [30]. The neuronal intrinsic properties in our study included spike threshold potential (Vts) and absolute refractory period (ARP). Vts were voltages of producing spikes [22,31,32]. ARPs were measured by injecting paired-depolarization pulses (3 ms) into the neurons after each spike (Figure 1). By altering inter-pulse intervals, we defined ARPs as the time from a complete spike to its subsequent one at 50% probability [33,34].

Data were analyzed if the recorded neurons had the resting membrane potentials negatively more than −60 mV, and action potential amplitudes more than 90 mV. The criteria for the acceptance of each experiment also included less than 5% changes in resting membrane potential, spike magnitude, and input resistance throughout each experiment. Input resistance was monitored by measuring cellular responses to hyperpolarization pulse at the same values as the depolarization that evoked action potentials. To estimate the effects of alkalosis versus acidosis on neuronal spikes and synaptic transmission, we measured sEPSC, sIPSC input-out curve, ISI, ARP and Vts under the conditions of control versus subsequent acidosis or of control versus subsequent alkalosis. We then calculated the differences of these values between alkalosis and control as well as between acidosis and control. The differences in sEPSCs versus their cumulative changes, sIPSCs versus their cumulative changes, input–output curve (stimulus intensities versus spikes), ISI, Vts and ARP were presented as mean ± SE. The comparisons of these parameters in the difference between alkalosis and control versus the difference between acidosis and control were done by t-test.

## Results

As alkalosis patients show severe neuropsychological deficit [11,12], we examined whether cortical neurons were more vulnerable to alkalosis than acidosis. In our studies, the functions of GABAergic neurons were evaluated under the conditions of control vs. alkalosis and control vs. acidosis. A use of GABAergic neurons to analyze cellular vulnerability was based on the facts that these cells were sensitive to pathological factors [6,8,13-15] and that they coordinated the activity of principal neurons [35,36]. The functions of GABAergic neurons in our analyses included their active properties, including their responsiveness to excitatory inputs, their capability to produce spikes and their outputs through inhibitory synapses.

Cellular alkalosis and acidosis were induced by changing pH environment onto the cortical slices from pH 7.35 to pH 8.0 or to pH 6.5 (Methods). With this protocol, the neuronal functions were recorded initially as a control and then were recorded under the conditions of alkalosis or acidosis. The components in these solutions were identical except for pH.

### Excitatory synaptic transmission on GABAergic neurons is more vulnerable to alkalosis than acidosis

**Figure 2:**
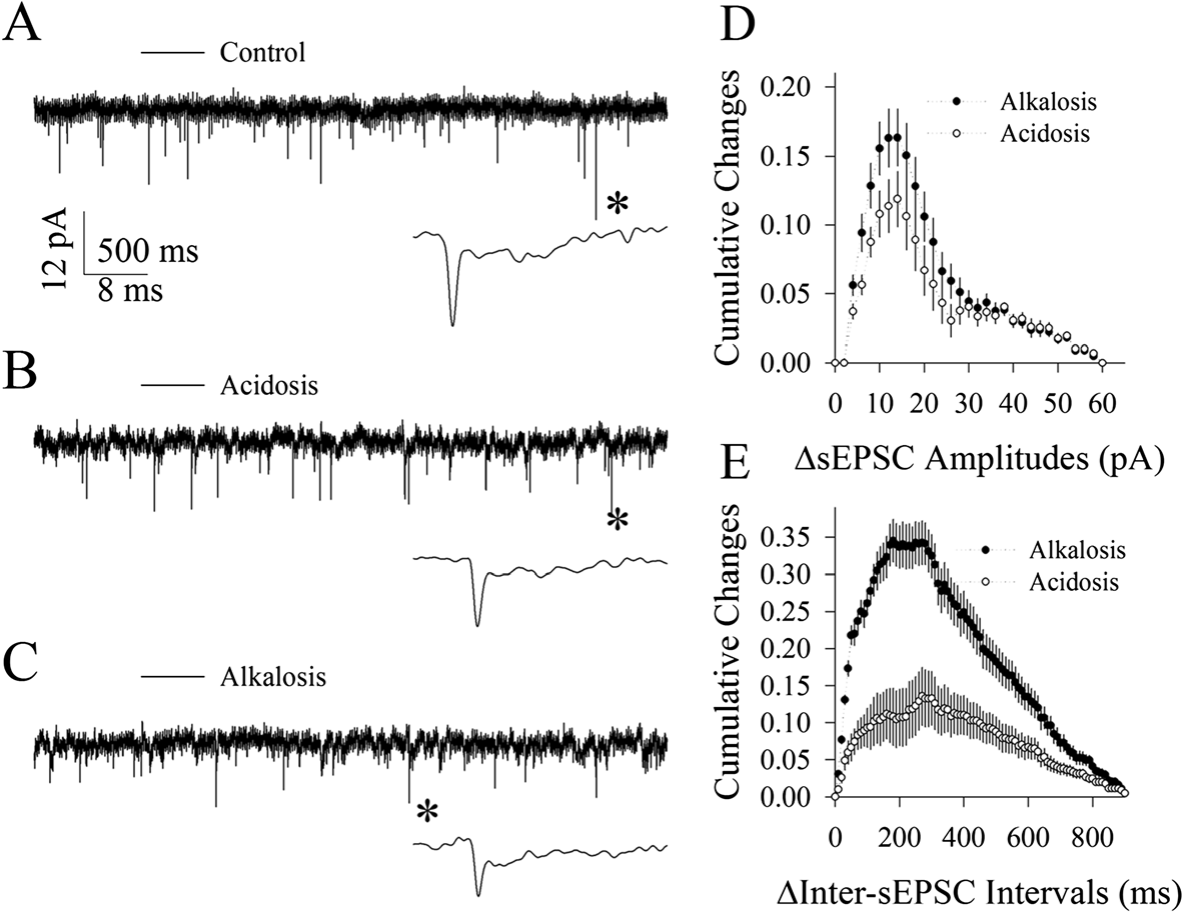
**Excitatory synaptic function at cortical GABAergic neurons is more vulnerable to alkalosis than acidosis. A~C)** Waveforms from top to bottom show the recorded sEPSCs under the conditions of control **(A)**, acidosis **(B)** and alkalosis **(C)**, respectively. The bottom waveforms from each of them are sEPSCs expanded from asterisk-indicated events. Calibration bars are 12 pA, 500 ms for top traces and 8 ms for the expanded traces. **D)** shows statistical comparisons in the net changes (Δ) of sEPSC amplitudes versus cumulative changes under the conditions of acidosis versus control (open symbols) and alkalosis versus control (dark symbols, p < 0.01; n = 12). **E)** shows the statistical comparison in the net changes (Δ) of inter-sEPSC-intervals versus cumulative changes under the conditions of acidosis versus control (open symbols) and alkalosis versus control (dark symbols, p < 0.01; n = 12).

Spontaneous excitatory postsynaptic currents (sEPSC) were recorded on GABAergic neurons by whole-cell voltage-clamp to assess the vulnerability of their synapses to alkalosis versus acidosis. sEPSCs recorded from each cell in a sequence from control to alkalosis or from control to acidosis were plotted as cumulative probability versus sEPSC amplitudes and inter-sEPSC-intervals [8]. Their values and curves were subtracted in control versus alkalosis as well as in control versus acidosis, in order to have cumulative changes versus sEPSC amplitudes (Figure 2D) and cumulative changes versus inter-sEPSC-intervals (Figure 2E). In given sEPSC amplitudes and inter-EPSC-intervals, their differences (Δ) between alkalosis and control and their differences between acidosis and control were compared. These ΔsEPSC amplitudes and intervals indicate their net changes, i.e., the influences of alkalosis or acidosis on synaptic transmission.

Figure 2 illustrates the comparisons in the effect of alkalosis on excitatory synaptic transmission with the effect of acidosis. Alkalosis, compared with acidosis, appears more obviously to attenuate sEPSC amplitudes and frequency (Figure 2A ~ C). Statistical data in Figure 2D illustrate the differences of sEPSC amplitudes in cumulative changes under the conditions of acidosis minus control (open symbols) as well as of alkalosis minus control (dark symbols; n = 12; p < 0.05 in a range of sEPSCs within 10 ~ 20 pA). Figure 2E illustrates Δinter-sEPSC-intervals in cumulative changes between acidosis and control (open symbols) and between alkalosis and control (dark symbol; n = 12; p < 0.01). Significant differences in the net changes of EPSCs by alkalosis than acidosis indicate that excitatory synaptic transmission on GABAergic cells is more vulnerable to alkalosis than acidosis. We then examined the influences of alkalosis and acidosis on their intrinsic properties.

### The ability of GABAergic neurons to encode spikes is more vulnerable to alkalosis than acidosis

Sequential spikes at cortical GABAergic neurons were induced by injecting depolarization pulses. Input–output curves (stimulus intensities versus spikes), inter-spike interval (ISI), spike refractory period (RP) and threshold potential (Vts) were measured, which reflected their active intrinsic ability to produce the spikes. The net changes of these parameters from control to alkalosis or from control to acidosis were calculated in each of the neurons for statistical average and comparison.

**Figure 3:**
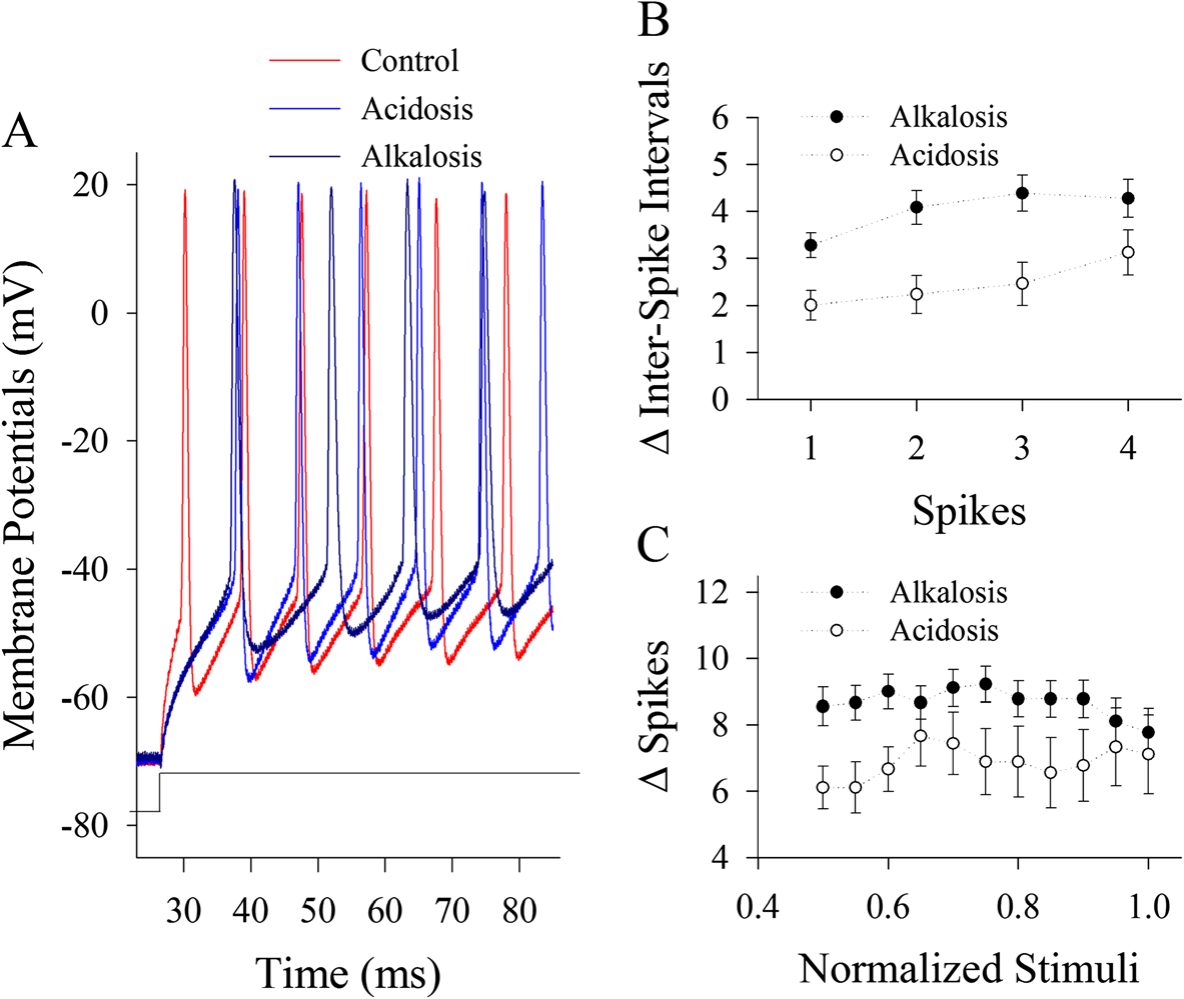
**The spike encoding processes of cortical GABAergic neurons are more vulnerable to alkalosis than acidosis. A)** Superimposed waveforms show sequential spikes induced by identical protocols under the conditions of control (red trace), acidosis (blue) and alkalosis (dark-blue). **B)** shows the statistical data in the net changes of ISI for spikes 1 ~ 2, 2 ~ 3, 3 ~ 4 and 4 ~ 5 under the conditions of acidosis minus control (open symbols) and alkalosis minus control (dark symbols, p < 0.05; n = 15). **C)** shows the statistical data in the net changes of spikes versus normalized stimuli under the conditions of acidosis minus control (open symbols) and alkalosis minus control (dark symbols, p < 0.05; n = 15).

Figure 3 shows the comparisons in the effects of alkalosis on input–output and ISI with the effects of acidosis. Spike frequencies appear to be lower in alkalosis (dark-blue trace in Figure 3A) than acidosis (blue). Statistical data in Figure 3B illustrates the net changes of inter-spike intervals that were calculated by acidosis minus control (open symbols) as well as alkalosis minus control (dark symbols, p < 0.05; n = 15). Figure 3C shows the net changes of normalized stimuli versus spikes that were the subtraction of acidosis to control (open symbols) as well as the subtraction of alkalosis to control (dark symbols, p < 0.05; n = 15). Significant net decreases in spike frequency by alkalosis than acidosis imply that the function of cortical GABAergic neurons is more vulnerable to alkalosis than acidosis.

Action potentials are navigated by refractory periods and threshold potential mediated by voltage-gated sodium channel [33,37,38]. Net changes in encoding spikes at GABAergic neurons are likely present in threshold potentials (Vts) and/or refractory periods (RP). ARPs and Vts were measured under the conditions from control to alkalosis or to acidosis.

Figure 1 illustrates the effects of alkalosis and acidosis on spike refractory periods and threshold potentials. Spike RPs appear longer in alkalosis (dark-blue traces in Figure 1A) than acidosis (blue traces). Figure 1B shows the net changes of RPs under the conditions of acidosis (gray bar) and alkalosis (dark-gray, an asterisk, p < 0.05; n = 15). Figure 1C shows ΔVts under the conditions of acidosis (gray bar) and alkalosis (dark-gray, p < 0.05; n = 15). The more net change of intrinsic properties induced by alkalosis than acidosis further supports that GABAergic neurons is more vulnerable to alkalosis than acidosis.

### GABAergic synapses are more vulnerable to alkalosis than acidosis

**Figure 4:**
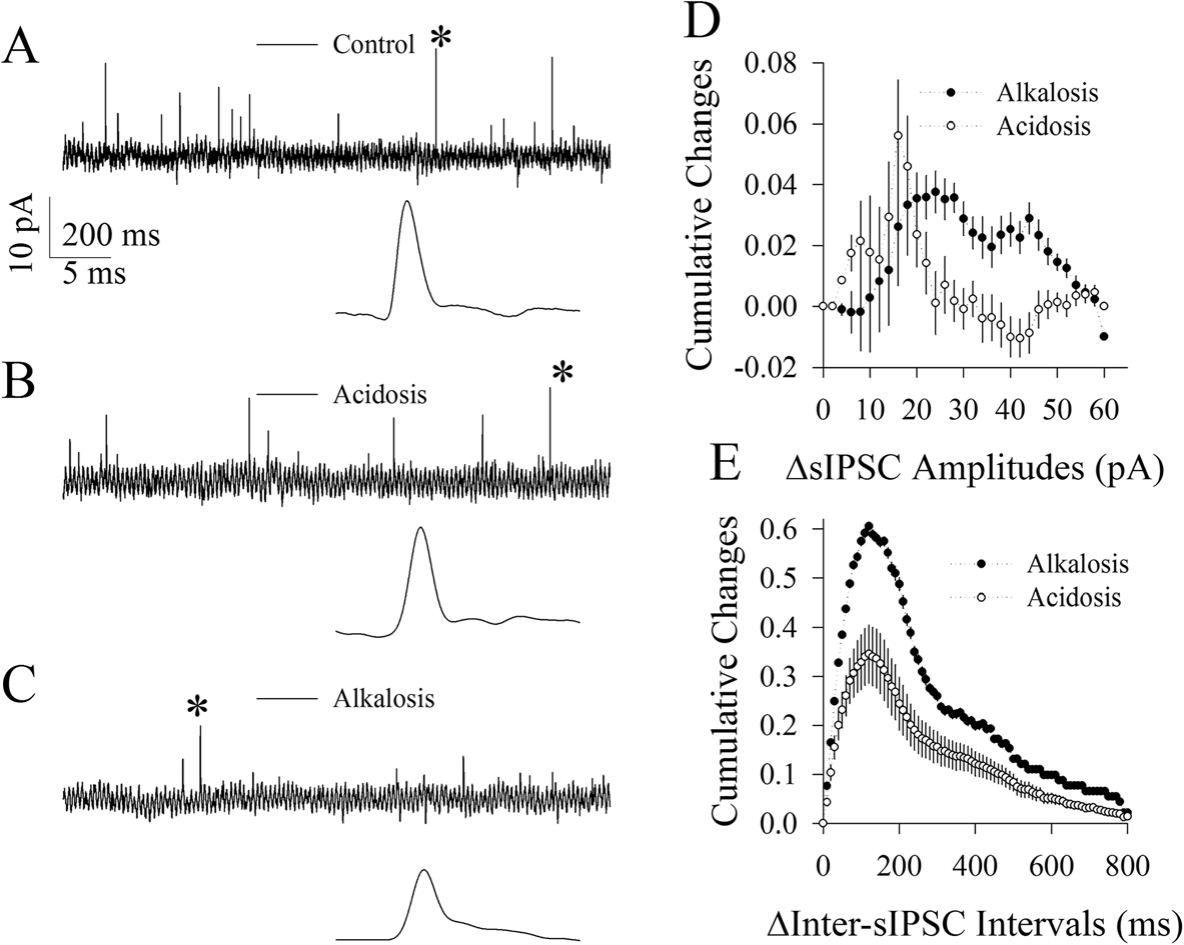
**Cortical GABAergic synapses are more vulnerable to alkalosis than acidosis. A~C)** Waveforms from top to bottom panels show the recorded sIPSCs under the conditions of control **(A)**, acidosis **(B)** and alkalosis **(C)**, respectively. The bottom waveforms from each of them are sIPSCs expanded from asterisk-indicated events. Calibration bars are 10 pA, 200 ms for top traces and 5 ms for the expanded traces. **D)** shows statistical comparison in the net changes (Δ) of sIPSC amplitudes versus cumulative changes under the conditions of acidosis versus control (open symbols) and alkalosis versus control (dark symbols, n = 10; p < 0.05). **E)** illustrates the statistical comparison in the net changes (Δ) of inter-sIPSC-intervals versus cumulative changes under the conditions of acidosis versus control (open symbols) and alkalosis versus control (dark symbols, p < 0.01; n = 10).

Spontaneous inhibitory postsynaptic currents (sIPSC) were recorded at pyramidal cells to assess the vulnerability of inhibitory synapses to alkalosis versus acidosis. Figure 4 illustrates the influences of alkalosis and acidosis on GABAergic synaptic activity. Alkalosis, compared with acidosis, appears more obviously to attenuate sIPSC amplitudes and frequency (Figure 4A ~ C). Statistical data in Figure 4D show the differences of sIPSC amplitudes in cumulative changes under the conditions of acidosis minus control (open symbols) as well as of alkalosis minus control (dark symbols; n = 10; p < 0.05 in a range of sIPSCs above 25 pA). Statistical data in Figure 4E show Δinter-sIPSC-intervals in cumulative changes between acidosis and control (open symbols) and between alkalosis and control (dark symbol; n = 10; p < 0.01). The significant net decreases in inhibitory synaptic functions by alkalosis than acidosis indicate that cortical GABAergic synapses are more vulnerable to alkalosis than acidosis.

## Discussion

Our study indicates that synaptic transmission and spike encoding related to the active function of cortical GABAergic neurons are more vulnerable to alkalosis than acidosis (Figures 1, 2, 3, 4), which explains well-known facts that patients suffered from alkalosis show severer brain dysfunction and that alkalosis-induced neuropsychological deficits are difficultly corrected, compared with those suffered from acidosis [11,12]. Vulnerable changes in our analyses include the amplitudes and frequencies of synaptic analogue signals and the capability of neuronal encodings in the digital spikes. The final molecular targets for alkalosis versus acidosis actions will be the voltage-gated sodium channels, glutamate receptors, GABA receptors and transmitter release machineries in cortical GABAergic neurons.

We first showed the differential vulnerability of GABAergic neurons to alkalosis and acidosis by analyzing the influence of alkalosis or acidosis on their responsiveness to excitatory synaptic inputs, their ability to produces spikes as well as their axonal output for inhibitory transmitter release. Compared with previous studies that the GABAergic neurons are sensitive to pathological factors [6,8,13-15], our analyses in the vulnerability of GABAergic neurons to acidosis vs. alkalosis appear to more precisely and specifically indicate that they are more vulnerable to pathogens for neurological disorders.

The molecular mechanisms underlying the more vulnerability of cortical neurons to alkalosis than acidosis remain elusive. The testable hypotheses include the different responses of cellular molecules to alkalosis and acidosis, such as intracellular signaling pathways, voltage-gated sodium channels, glutamate receptor-channels, GABA receptor-channels and presynaptic proteins related to neurotransmitter release. In order to address this issue, certain data obtained from studying other types of cells may be referred. For example, alkalinization and acidification regulate cell functions differently [39]. The cellular buffers favor to balance acidosis such that the cells are more suffered in alkalosis than acidosis [40]. Compared with acidosis, alkalosis was found to more obviously influence blood vessel resistance [41], vascular proton-ATPase expression [42] and cellular functions [43].

In terms of clinical benefit from our study, our thoughts are given below. As GABAergic neurons are more vulnerable to pathological factors, the treatment of neurological disorders would be benefit from securing the function of GABAergic cellular units. Furthermore, the neurological disorders caused by the imbalance of acidosis and alkalosis should be focused on protecting the functions of GABAergic neurons.

## Conclusion

The severer impairment of cortical GABAergic neurons in alkalosis patients leads to more critical neural excitotoxicity, such that alkalosis-induced brain malfunction will be difficultly corrected, compared to acidosis. The vulnerability of cortical GABAergic neurons to high pH is likely a basis of severe clinical outcomes in alkalosis versus acidosis.

## Competing interests

The authors declare that they have no competing interests.

## Authors’ contributions

SZ and PS designed project and wrote manuscript. SZ, PS, ZS, JZ, JZ and YG conducted experiments and data analyses. All authors read and approved the final manuscript.

## Pre-publication history

The pre-publication history for this paper can be accessed here:

http://www.biomedcentral.com/1471-2377/13/192/prepub
